# Closha: bioinformatics workflow system for the analysis of massive sequencing data

**DOI:** 10.1186/s12859-018-2019-3

**Published:** 2018-02-19

**Authors:** GunHwan Ko, Pan-Gyu Kim, Jongcheol Yoon, Gukhee Han, Seong-Jin Park, Wangho Song, Byungwook Lee

**Affiliations:** Korean BioInformation Center (KOBIC), KRIBB, 125 Gwahangno, Yuseong-gu, Daejeon, 34141 South Korea

## Abstract

**Background:**

While next-generation sequencing (NGS) costs have fallen in recent years, the cost and complexity of computation remain substantial obstacles to the use of NGS in bio-medical care and genomic research. The rapidly increasing amounts of data available from the new high-throughput methods have made data processing infeasible without automated pipelines. The integration of data and analytic resources into workflow systems provides a solution to the problem by simplifying the task of data analysis.

**Results:**

To address this challenge, we developed a cloud-based workflow management system, Closha, to provide fast and cost-effective analysis of massive genomic data. We implemented complex workflows making optimal use of high-performance computing clusters. Closha allows users to create multi-step analyses using drag and drop functionality and to modify the parameters of pipeline tools. Users can also import the Galaxy pipelines into Closha. Closha is a hybrid system that enables users to use both analysis programs providing traditional tools and MapReduce-based big data analysis programs simultaneously in a single pipeline. Thus, the execution of analytics algorithms can be parallelized, speeding up the whole process. We also developed a high-speed data transmission solution, KoDS, to transmit a large amount of data at a fast rate. KoDS has a file transfer speed of up to 10 times that of normal FTP and HTTP. The computer hardware for Closha is 660 CPU cores and 800 TB of disk storage, enabling 500 jobs to run at the same time.

**Conclusions:**

Closha is a scalable, cost-effective, and publicly available web service for large-scale genomic data analysis. Closha supports the reliable and highly scalable execution of sequencing analysis workflows in a fully automated manner. Closha provides a user-friendly interface to all genomic scientists to try to derive accurate results from NGS platform data. The Closha cloud server is freely available for use from http://closha.kobic.re.kr/.

## Background

With the emergence of next generation sequencing (NGS) technology in 2005, the field of genomics is caught in a data deluge. Modern sequencing platforms are capable of sequencing approximately 5000 M-bases per day [1]. DNA sequencing is becoming faster and less expensive at a pace far outstripping Moore’s law, which describes the rate at which computing becomes faster and less expensive. As a result of the increased efficiency and diminished cost of NGS, the demand for clinical and agricultural applications is rapidly increasing [2]. In the bioinformatics community, acquiring massive sequencing data is always followed by large-scale computational analysis to process the data and obtain scientific insights. Therefore, investment in a sequencing instrument would normally be accompanied by substantial investment in computer hardware, analysis pipelines, and bioinformatics experts to analyze the data [3].

When genomic datasets were small, they could be analyzed on personal computers in a few hours or perhaps overnight [4]. However, this approach does not apply to large NGS datasets. Instead, researchers require high-performance computers and parallel algorithms to analyze their big genomic data in a timely manner [5]. While high-performance computing is essential for data analysis, only a small number of biomedical research labs are equipped to make effective and successful use of parallel computers [6]. Obstacles include the complexities inherent in managing large NGS datasets and assembling and configuring multi-step genome sequencing pipelines, as well as the difficulties inherent in adapting pipelines to process NGS data on parallel computers [7].

The difficulties in creating these complicated computational pipelines, installing and maintaining software packages, and obtaining sufficient computational resources tend to overwhelm bench biologists and prevent them from attempting to analyze their own genomic data [8]. Despite the availability of a vast set of computational tools and methods for genomic data analysis [1], it is still challenging for a genomic researcher to organize these tools, integrate them into workable pipelines, find accessible computational platforms, configure the computing environment, and perform the actual analysis.

To address these challenges, the MapReduce [9] model and the corresponding Apache Hadoop framework have been widely adopted to handle large data sets using parallel processing tools [10]. The most widely used open-source implementation of the MapReduce programming model for big data batch processing is Apache Hadoop. A cloud-based bioinformatics workflow platform has also been proposed for genomic researchers. Scientific workflow systems such as Galaxy [11] and Taverna [12] offer simple web-based workflow toolkits and scalable computing environments to meet this challenge.

Such efforts have resulted in significant insight into the technical requirements to leverage cloud computing for the analysis of genomic data [7], but problems still remain to be solved. Even though many applications have been developed for the analysis of genomic data, they are either tools running only on a MapReduce platform such as Hadoop BAM [13] or Crossbow [14] or general-purpose (mainly Linux-based) programs such as bowtie [2] and bwa [15]. It is crucial to integrate these two types of platform-based applications on a single pipeline. Transferring these big data is another problem, as NGS genomic data is too large to use cloud computing platform services [16].

We developed an automatic workflow management system, Closha, to provide a pipeline-based analysis service for massive biological data, especially NGS genomic data. Closha was developed as a hybrid system that can run both Hadoop-based and general-purpose applications on a single analysis pipeline. We also developed a high-speed data transmission solution, KoDS, to transmit a large amount of data at a fast rate. Closha makes it simple to create multi-step analysis using a simple drag and drop functionality. Using Closha, programs can be added and connected to each other so that the output of one program becomes the input of other programs. Our cloud-based workflow management system can help users to run in-house pipelines or construct a series of steps in an organized way.

## Methods

### Goals of Closha

The following three objectives drive the development of Closha. First, Closha seeks to increase access to intricate computational analyses for all genomic researchers, including those with limited or no programming knowledge. Our web-based graphical user interface (GUI) makes it simple to do everything needed for relatively large data analyses. Second, the Closha GUI provides a workflow editor in which users can simply create automated, multi-step analysis pipelines using drag and drop. Here, workflows refer to structured procedures that help users construct a series of steps in an organized way. Each step is a specific parametrized action that receives input and produces output. The analysis pipelines on Closha are exactly reproducible, and all analysis parameters and inputs are permanently recorded. Lastly, Closha enables users to share their pipelines on the web.

### Cluster configuration

**Fig. 1:**
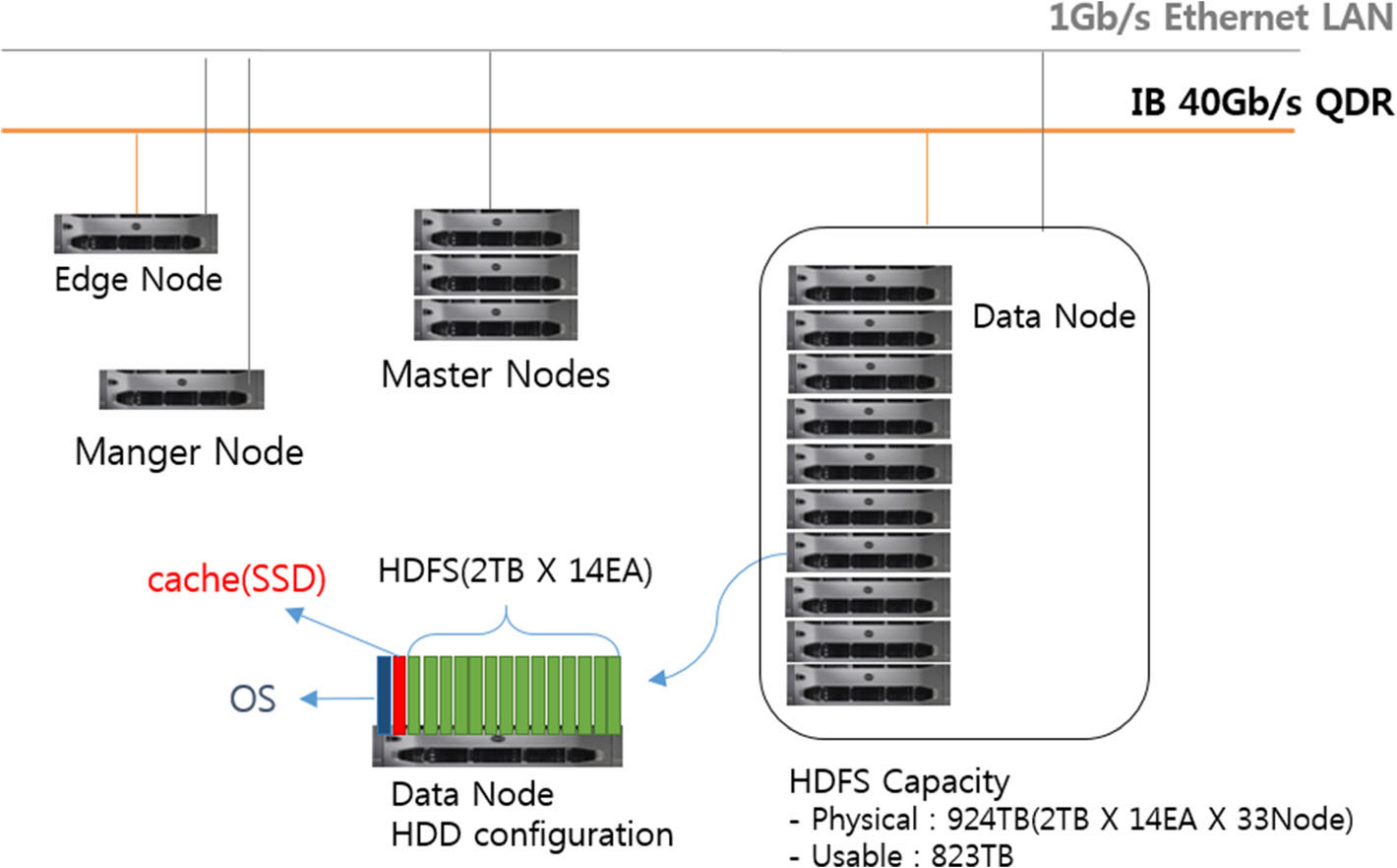
The architecture of the Closha system. The Closha system consists of distributed computing nodes: the master node (name node), slave nodes (data node), edge nodes, and node manager

All runs of analysis pipelines on Closha are performed on a cluster of five master nodes and 33 data (slave) nodes (Fig. 1). The Closha hardware system consists of 660 core CPUs, 2 TB of memory, and 800 TB of disk storage in total. Each node has an Intel Xeon E502690 v2 3.0 GHz CPU, 96 GB of memory, and 28 TB of disk storage. The data node HDD configuration consists of the Hadoop Distributed File System (HDFS) and a solid state drive (SSD) cache. HDFS is the primary distributed storage used by Hadoop applications. An SSD is a flash-based storage drive that is many times faster than a traditional hard drive, so using an SSD in the data node makes it possible to run Linux-based programs on the Hadoop cluster system. Edge nodes (gateway nodes) are the interface between the Hadoop cluster and the outside network. The edge nodes are commonly used to run client applications and cluster administration tools. The node manager (NM) handles the individual data nodes in a Hadoop cluster.

### Closha workspace

**Fig. 2:**
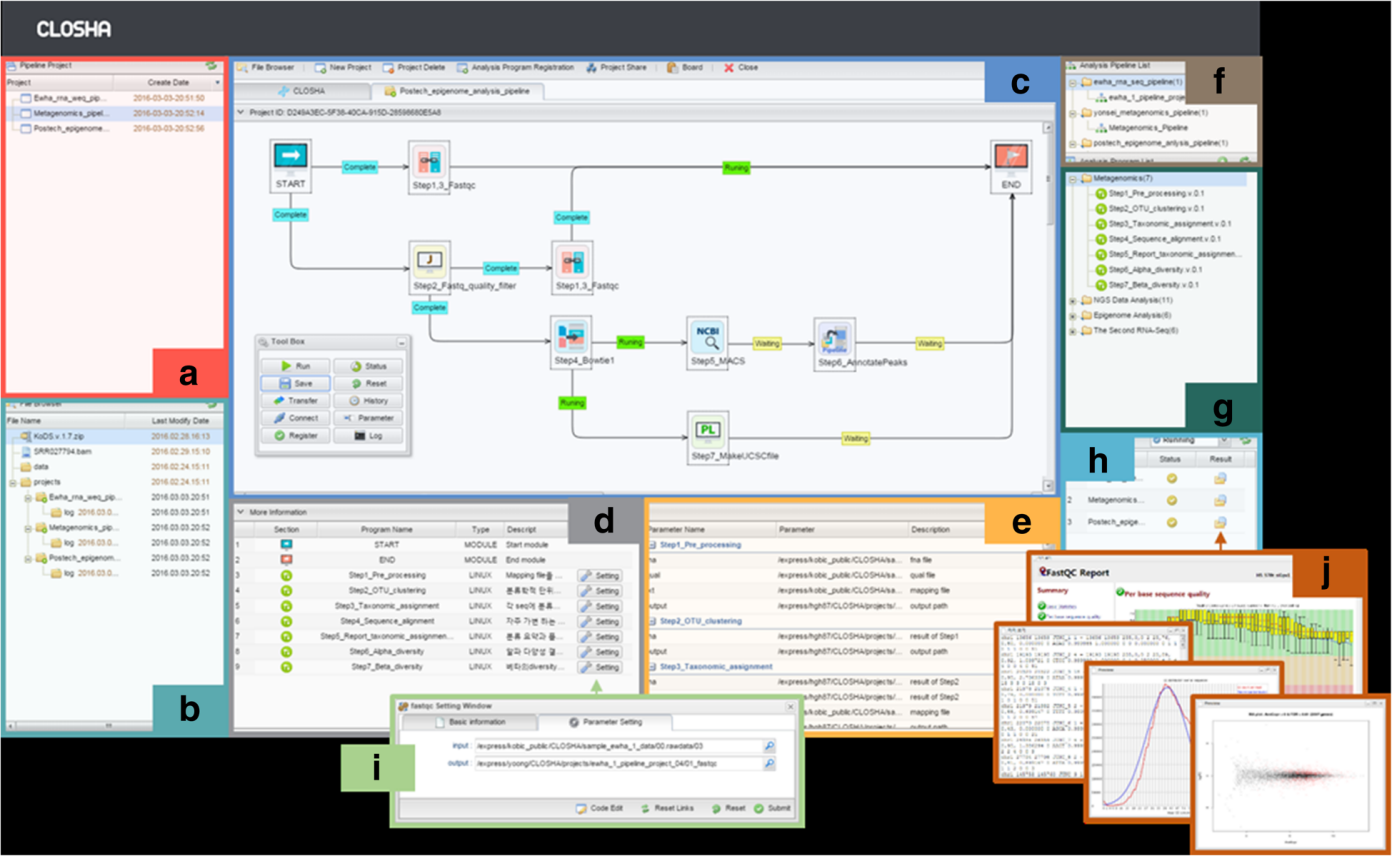
The interface of the Closha workspace. The web-based Closha workflow editor has several panels: **a** the pipeline project list, (**b**) the file explorer, (**c**) the canvas: pipeline modeling screen, (**d**) a table detailing the analysis program, (**e**) a table detailing the analysis program parameters, (**f**) the analysis pipeline list, (**g**) the list of analysis programs available for use, and (**h**) the pipeline project job execution history and current progress

The Closha GUI workspace is divided into eight panels that show information on the user’s projects, the file explorer, the pipeline modelling screen (canvas), the analysis programs (program panel), the analysis program parameters, the analysis pipeline list (pipeline panel), the list of analysis programs available for use, and the job execution history and current progress (execution and history panel) (Fig. 2).

Analysis pipelines are grouped into categories and can be searched on the pipeline panel. When a pipeline is selected, it is shown in the main window, where its parameters are set and the tool is executed. When a user executes a tool, its output datasets are added to the execution and history panel. The colors on the execution panel shows the state of tool execution. Clicking on a dataset in the panel provides a wealth of information, including the tool and parameter settings used to create it.

### Workflow editor (canvas)

The canvas is an interface for creating and modifying workflows (analysis pipelines) by arranging and connecting activities to drive processes. The canvas provides the working surface for creating new workflows or editing existing ones. Users can create custom workflows or use existing workflows on the screen. The canvas (Fig. 2) makes it simple to create multi-step analyses using drag and drop functionality. Using the canvas, existing and user-uploaded tools can be added and connected so that the output of one tool becomes the input of other tools. Tool parameters can be set in the parameter panel. Workflows enable the automation and repeated running of large analyses. Once created, workflows function as tools. They can be accessed and run from Closha’s main analysis interface.

### Representing analysis pipelines of workflows

The workflows in the analysis pipelines are commonly depicted as directed acyclical graphs, in which each of the vertices (modules or programs) has a unique identifier and represents a task to be performed. Additionally, each of the tasks in a workflow can receive inputs and can produce outputs. The outputs of a task can be directed through another task as input. An edge (connector) between two vertices represents the channeling of an output from one task into another. Edges determine the logical sequence. A task can be executed once all of its inputs can be resolved.

### Uploading data to Closha

**Fig. 3:**
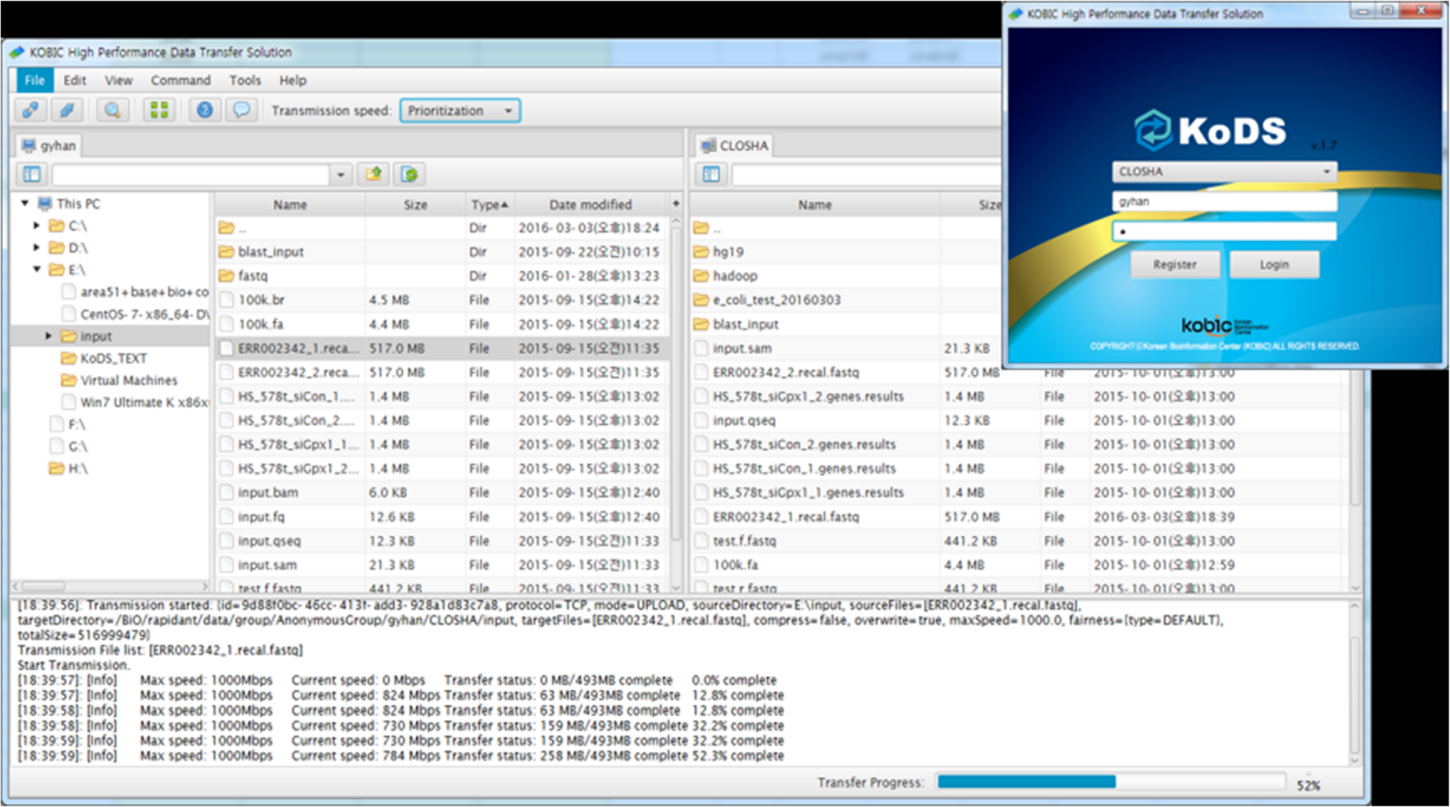
Screenshot of the KoDS tool. The left window is the user’s local computer and the right is the Closha server

We developed a fast file transfer tool, called KoDS, for uploading massive genomic data such as exome and RNA-Seq (RNA sequencing) data to the Closha server from the user’s local computer and for downloading the resulting files to the local computer (Fig. 3). The client program of KoDS can be downloaded from the Closha website and be installed on the user’s computer. The KoDS transfer platform provides users with secure high-speed movement of all of their data, supporting a wide range of server, desktop and Linux operating systems. Using KoDS, users can simultaneously upload an unlimited number of files to Closha. KoDS has a file transfer speed up to 10 times that of normal FTP and HTTP protocols.

### Hybrid system

We implemented a service-oriented architecture, a hybrid system, to allow arbitrary tools to be described as services. The hybrid system provides access to traditional applications on a cloud infrastructure, which enables users to use both the MapReduce tools and the traditional programs in a single pipeline simultaneously. Thus, the execution of analytical algorithms can be parallelized, speeding up the whole process.

### Elastic scalability

Scalability is the capability of a system, network, or process to handle a growing amount of work or its potential to be enlarged to accommodate that growth. For example, a system is considered scalable if it can increase its total output under an increased load when resources (typically hardware) are added. A system whose performance improves after adding hardware, in proportion to the capacity added, is said to be a scalable system. Scalability is one of the most attractive prospects of the benefit-rich phenomenon of cloud computing and provides a useful safety net for when a user’s needs and demands change. The resource manager and the job controller on Closha elastically control the scalability by either increasing or decreasing the required resources.

## Results

### Analysis pipelines

As of October 1st, approximately 200 analysis tools were installed on Closha, and 20 analysis pipelines were available for the analysis of exome, RNA-Seq, and ChiP-Seq, data, among others. Closha has two types of pipelines: registered and new. Users can use a registered pipeline suitable for their genomic data by selecting a pipeline in the Closha analysis pipeline list. If users want to create a new analysis pipeline, they can build their own pipeline either from scratch or by modifying a registered pipeline with installed or user-defined tools.

### RNA-Seq pipeline

**Fig. 4:**
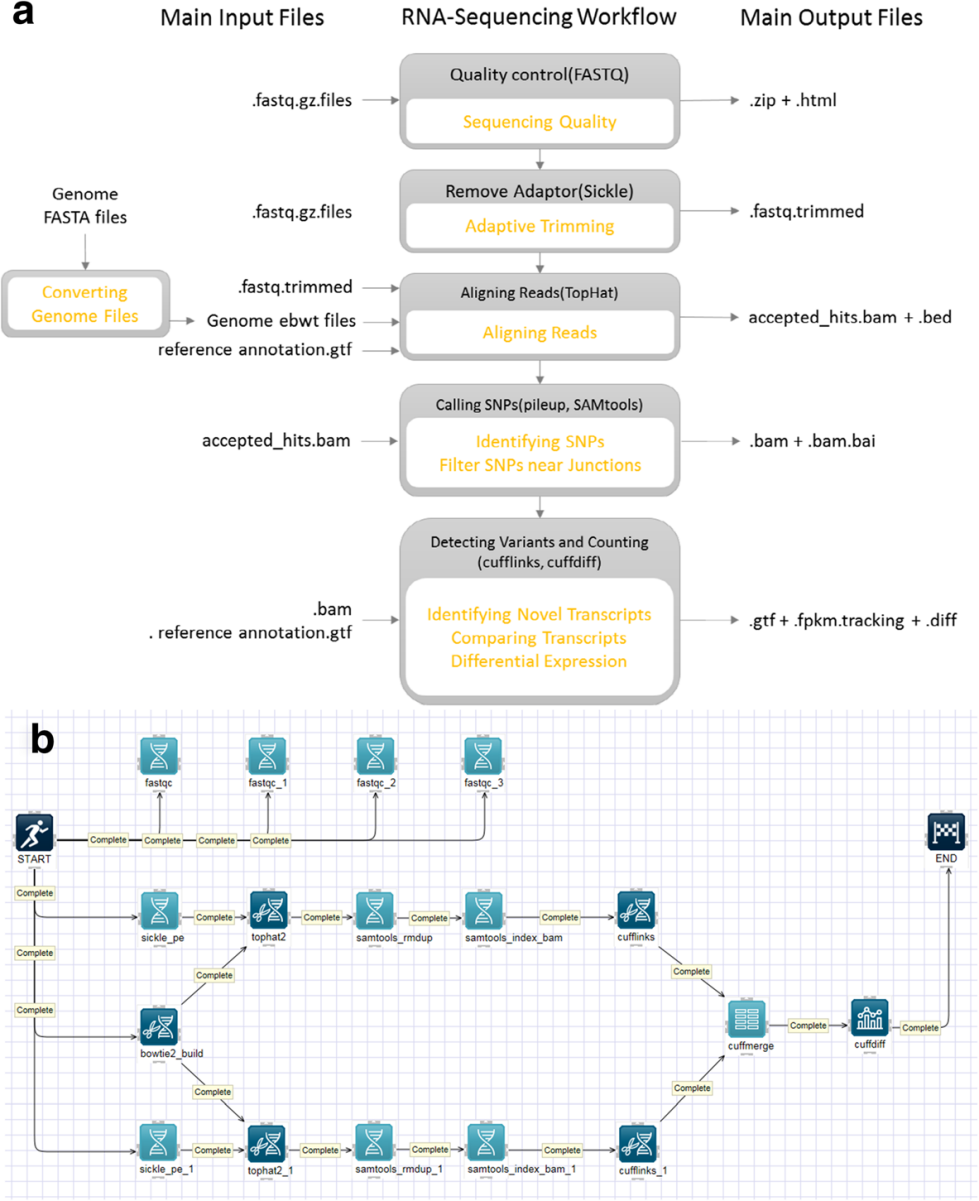
Screenshot of the RNA-Seq schematic diagram and its pipeline. **a** Schematic overview of the RNA-Seq pipeline. **b** The RNA-Seq pipeline implemented on the Closha canvas

We use a representative NGS analysis workflow of RNA-Seq to examine the time and cost of execution on Closha cloud configurations. RNA-Seq is a deep-sequencing technique used to explore and profile the entire transcriptome of any organism. Analyzing an organism’s transcriptome is important for understanding the functional elements of a genome. We built an RNA-Seq analysis pipeline in which we use the KoDS tool to move data from a local machine to the Closha server. Figure 4a shows a schematic overview of the RNA-Seq pipeline. Then, we can obtain the resulting output data at the end of the pipeline.

The pipeline includes five analysis tools: TopHat [17], Cufflinks, Cuffmerge, Cuffdiff, and limma voom [18]. TopHat is a fast splice junction mapper that is used to align RNA-Seq reads to large genomes and analyze the mapping results to identify splicing junctions between exons. TopHat internally uses the Bowtie tool, an ultra-high-throughput short read aligner. Cufflinks is used to assemble these alignments into a parsimonious set of transcripts and then estimate the relative abundances of these transcripts. The main purpose of Cuffmerge is to merge several Cufflinks assemblies, making it easier to produce an assembly GTF file suitable for use with Cuffdiff. Cuffdiff is then used to find significant changes in transcript expression, splicing, and promoter use. Finally, voom robustly estimates the mean-variance relationship and generates a precision weight for each individual normalized observation. It can be used to calculate differently expressed genes (DEGs) from the transcript expression level. Figure 4b depicts the implemented RNA-Seq pipeline on the Closha canvas.

To evaluate the execution of the RNA-Seq pipeline in Closha, we used an RNA-Seq case-control sample data set: 42,112,235 paired-end case reads and 40,975,645 paired-end control reads. The total sample size of the case and the control reads is approximately 42GB. The execution of the RNA-Seq DEG pipeline on the case and the control data provided the baseline runtime speed. Closha assigned four CPU cores and 16GB of memory for a single RNA-Seq job. The execution of the RNA-Seq pipeline on the sample data using Closha takes a total of 3 h 44 mins and most of the time was spent on the running of the TopHat2 program (2 h 36 mins). We performed a comparison experiment between Closha and Galaxy with the same data and the same RNA-Seq pipeline. The same machine was used for the comparison. The execution time using Galaxy was 6 h 11 mins, showing Closha has approximately 1.7 times better performance than Galaxy in the execution of the RNA-Seq pipeline (Table 1).

**Table 1:** Execution time of each program of Closha and Galaxy in the RNA-Seq analysis

Analysis steps (programs)	Running time	
	Closha	Galaxy
Data transfer	9 mins	1 h 14 mins
FastQC	3 mins	5 mins
Sickle	3 mins	11 mins
TopHat2	2 h 36 mins	3 h 4 mins
SAMtools	13 mins	15 mins
Cufflinks	10 mins	16 mins
Cuffdiff and voom	30 mins	1 h 6 mins
	Total running time: 3 h 44 mins	Total running time: 6 h 11 mins

To simulate real use with multiple executions, we performed batched jobs of the example data simultaneously, scaling up by adding 100 jobs of the sample data. We found little change in execution time as the number of batched jobs increased, which means that the Closha cloud system can run an RNA-Seq pipeline of up to 500 jobs at the same time with little change in execution time (Table 2).

**Table 2:** Running time of multiple jobs

No. of jobs	100	200	300	400	500
Running time of each job	3 mins 44 s	3 mins 59 s	3 mins 53 s	3 mins 42 s	3 mins 58 s

### Creating a new pipeline

Closha allows users to create their own pipelines to analyze their own data on the canvas. To create a new analysis pipeline, users click the ‘New Pipeline’ button in the top menu of Closha, enter the name and description of the pipeline, and select an analysis pipeline type. Users will have only the [Start] and [End] modules on the canvas immediately upon creating a pipeline after selecting a ‘new analysis pipeline design’ in the project type. Users can drag and drop their desired analysis programs in the list of analysis programs on the right of the canvas. Upon positioning a desired analysis program on the canvas, when the users places the mouse over the edge of the analysis program icon, a connection mark will be created that can be drawn to the module. Starting from the mark, the connector must be dragged until the icon of the next analysis program to be connected turns translucent. Users can make connections to the start module, the analysis program and the end module using this method to perform the analysis.

Then, users can set the parameter values by clicking the ‘Set Parameters’ button on the toolbar before executing the pipeline project. On the creation of an initial project, default parameter values are automatically assigned. Users can change the parameter values in accordance with the conditions required to set and analyze their input data. To connect user files to Closha, the user can click the ‘File Selection’ icon in the field to open a window that allows the selection of an input file and then a personal or common-use data and the desired file in the file list. The path for the output file is automatically a sub path of the project in setting the input data. Finally, the analysis pipeline is executed with a message that the analysis has started. The status of the project is displayed on a real-time basis in three modes: Complete, Execute, and Wait.

**Fig. 5:**
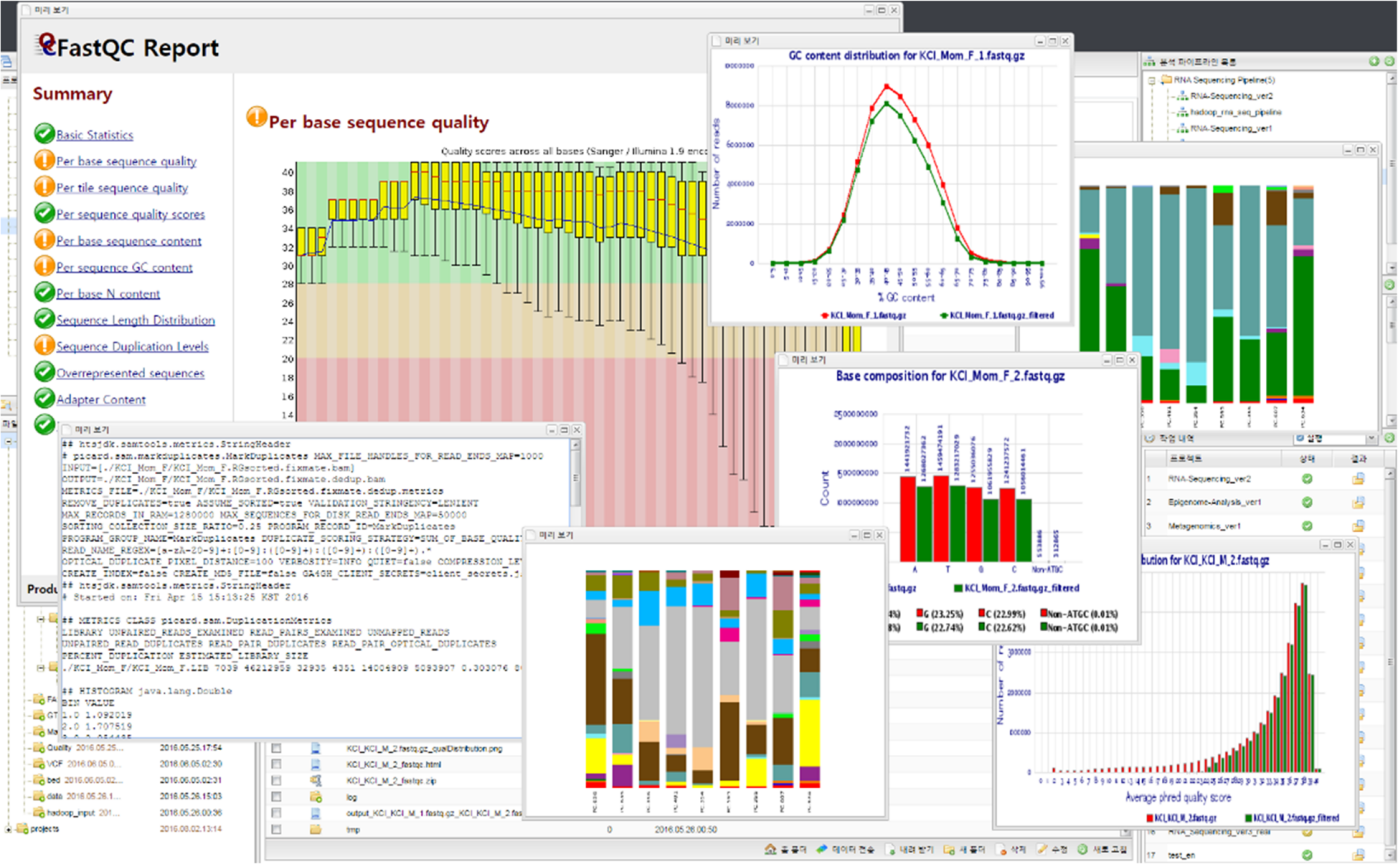
Screenshot of Closha results files. Closha allows users to view files in various formats, including text, HTML, and PNG on the web without having to download the files

Users can see the results files by clicking the ‘Result’ icon on the menu bar and downloading them to the local computer by clicking the ‘Download’ button in the bottom menu, which allows KoDS to be used for high-speed transmission. Closha also allows users to view files in various formats including text, HTML, and PNG on the screen without having to download the files (Fig. 5).

## Discussion

The Closha computing service is an attractive, efficient and potentially cost-effective alternative for the analysis of large genomic datasets. Closha offers a dynamic, economical, and versatile solution for large-scale computational analysis. Our work on genomic data demonstrate that Closha implementation provides a scalable, robust and efficient solution to address the ever-increasing demand for efficient genomic sequence analysis. Closha allows genomic researchers without informatics or programming expertise to perform complex large-scale analysis with only a web browser. Its potentials for computing with NGS genomic data could eventually revolutionize life science and medical informatics.

## Conclusions

We developed a cloud-based workflow management system to provide fast and cost-effective analysis of massive genomic data. We implemented complex workflows making optimal use of high-performance computing clusters. Closha allows users to create multi-step analyses using drag and drop functionality and to modify the parameters of pipeline tools. We also developed a high-speed data transmission solution to transmit a large amount of data at a fast rate. KoDS has a file transfer speed of up to 10 times that of normal FTP and HTTP. The computer hardware for Closha is 660 CPU cores and 800 TB of disk storage, enabling 500 jobs to run at the same time. Closha is a scalable, cost-effective, and publicly available web service for large-scale genomic data analysis. Closha supports the reliable and highly scalable execution of sequencing analysis workflows in a fully automated manner. Closha provides a user-friendly interface to all genomic scientists to try to derive accurate results from NGS platform data.
